# Vaccine candidates based on MVA viral vectors expressing VP2 or VP7 confer full protection against Epizootic hemorrhagic disease virus in IFNAR(−/−) mice

**DOI:** 10.1128/jvi.01687-24

**Published:** 2024-11-07

**Authors:** Luis Jiménez-Cabello, Sergio Utrilla-Trigo, Karen Rodríguez-Sabando, Alejandro Carra-Valenzuela, Miguel Illescas-Amo, Eva Calvo-Pinilla, Javier Ortego

**Affiliations:** 1 Centro de Investigación en Sanidad Animal (CISA), Instituto Nacional de Investigación y Tecnología Agraria y Alimentaria (INIA), Valdeolmos, Madrid, Spain; University of Michigan Medical School, Ann Arbor, Michigan, USA

**Keywords:** Epizootic hemorrhagic disease virus, IFNAR(-/-) mouse, vaccine, MVA, VP2, VP7, multiserotype, DIVA

## Abstract

Epizootic hemorrhagic disease (EHD), caused by Epizootic hemorrhagic disease virus (EHDV), is an emerging and severe livestock disease. Recent incursion and distribution of EHDV in Europe have outlined the need for vaccine research against this viral disease. In this work, we report modified vaccinia virus Ankara (MVA)-vectored vaccines designed to express protein VP2 of EHDV-8 or protein VP7 of EHDV-2. Prime boost immunization of adult IFNAR(−/−) mice with the MVA-VP2 vaccine candidate induced high titers of EHDV-8-specific neutralizing antibodies (NAbs) and conferred full protection against homologous lethal challenge with EHDV-8. However, no heterologous protection was observed after lethal challenge with EHDV-6. In contrast, the MVA-VP7 vaccine candidate elicited strong cytotoxic CD8+ T-cell responses against VP7 and conferred complete protection against lethal challenge with either EHDV-8 or EHDV-6 in IFNAR(−/−) mice in the absence of NAbs, being the first multiserotype vaccine candidate against EHDV. Moreover, we expressed recombinant proteins VP2 and VP7 of EHDV in the baculovirus expression system, which were used to analyze the potential DIVA (differentiating infected from vaccinated animals) character of these vaccine candidates.

**IMPORTANCE:**

Emergence and re-emergence of arthropod-borne viruses are major concerns for both human and animal health. The most recent example is the fast expansion of EHDV-8 through Europe. Besides, EHDV-8 relates with a high prevalence of pathologic cases in cattle populations. No vaccine is currently available in Europe, and vaccine research against this arboviral disease is negligible. In this work, we present novel DIVA vaccine candidates against EHDV, and most importantly, we identified the protein VP7 of EHDV as an antigen capable of inducing multiserotype protection, one of the major challenges in vaccine research against orbiviruses.

## INTRODUCTION

Epizootic hemorrhagic disease (EHD) is a non-contagious arthropod-borne disease that affects both wild and domestic ruminants, with cervids and specially white-tailed deer (WTD) (*Odocoileus virginianus*), as the most affected hosts (1, 2). It is included in both USA’s National List of Reportable Animal Diseases and the notifiable disease list of the World Organization for Animal Health (WOAH) (3, 4). Epizootic hemorrhagic disease virus (EHDV), an arbovirus that belongs to the genus *Orbivirus* within the family *Sedoreoviridae*, is the etiological agent of this disease. Alike the highly related bluetongue virus (BTV), EHDV is a non-enveloped virus characterized by its icosahedral capsid (∼80 nm of diameter) composed of an outer layer formed by proteins VP2 and VP5, an intermediate layer constituted by VP7 and inner capsid composed by VP3, inside of which are the genome segments and copies of three proteins with enzymatic properties that form the replication complex: VP1, VP4, and VP6 (5–11). The genome, composed by 10 segments of double-stranded RNA (dsRNA), also encodes for five non-structural proteins (NS1–NS5) (5, 12–18). To date, at least seven serotypes (EHDV-1, 2, 4, 5, 6, 7, and 8) have been identified, and more putative serotypes are being described (19–22). Outer capsid protein VP2 is the most variable protein among EHDV serotypes (19). Like BTV, VP2 accomplishes key roles during early stages of infection, being involved in virus attachment and entry into host cells, and is the main inducer of virus-neutralizing antibodies (NAbs) (23). In consequence, it defines virus serotype based on cross-neutralization assays and extensive phylogenetic studies (19).

EHDV is distributed worldwide (1). Due to climate and anthropogenic factors, outbreaks of EHDV occur in regions where the virus was not present before. In October 2022, EHDV-8 was detected for the first time in Europe (24). The arrival of EHDV-8 in Italy and Spain ended up in widespread distribution in these countries. Moreover, new outbreaks of EHDV-8 were declared in Portugal and France in October 2023, and propagation to other European EHDV-free countries may occur (25). Traditionally, EHDV causes negligible mortality and low morbidity in cattle compared with those observed in deer, with the exception of Ibaraki strain (EHDV-2) (26, 27). Nonetheless, recent outbreaks have been characterized by an increased pathogenicity in cattle populations (28). It is assumed that EHDV causes significant economic losses in the livestock industry, due to the reduction of milk and meat production, infertility, or abortion. In this sense, so far, only one assessment of economic losses has been reported and corresponds to the EHDV-7 outbreak that occurred in Israel during the fall of 2006, with an estimated loss ranging from USD 1,591,000 to USD 3,391,000 (29).

Due to the expansion of EHDV, its increased pathogenicity in livestock and the lack of therapeutic treatments (30), development of effective vaccines is paramount. Vaccines based on conventional approaches have been developed against EHDV. In Japan, two vaccines against EHDV-2 are commercially available: a monovalent live attenuated vaccine (LAV) and an inactivated bivalent vaccine (against EHDV-2 and bovine ephemeral fever, caused by bovine ephemeral fever virus) (31). In the USA, autogenous vaccines have been used (32). However, LAVs imply some disadvantages, such as reversion to virulence (33), transmission to insect vectors (34–36), genetic reassortment between wild-type viruses and vaccine strains (33, 37), or under attenuation (34, 38). Inactivated vaccines are relatively safe but less immunogenic than LAVs, requiring booster doses (39). Besides, both approaches do not permit to differentiate serologically between infected and vaccinated animals (DIVA) and do not confer cross-protection between serotypes.

Next-generation vaccines are developed to avoid the disadvantages of conventional vaccine approaches. For EHDV, protein VP2 has been the main antigen considered for vaccine development as it is the target of NAbs. Indeed, the unique recombinant vaccine candidate evaluated against EHDV consisted of recombinant protein VP2 of serotypes 2 or 6, showing high immunogenicity and efficacy to confer protection in WTD (40). Virus like-particles (VLPs) have been also generated for different serotypes of EHDV, showing immunogenicity in laboratory animal models although their potential to confer protection against EHDV has not been evaluated (41). Viral vector vaccines have been extensively developed for BTV vaccine research (42). Viral vectors are highly immunogenic, being able to induce potent and long-lasting humoral and cellular immune responses (43–45). Among them, modified vaccina virus Ankara (MVA) has been widely used as a viral vaccine vector due to its stability, safety, ability to deliver foreign genes, and capacity to induct strong T and B-cell immune responses (43, 44, 46–48).

As stated above, VP2 is characterized by its high variability among EHDV serotypes, being the target of serotype-specific NAbs (32). For generation of multiserotype vaccine approaches against EHDV, highly conserved EHDV proteins should be explored. In this sense, the serogroup-specific protein VP7 is highly conserved (98%) between the different EHDV serotypes (49, 50). Although the potential of this EHDV antigen for inducing protection against this disease has not been elucidated yet, immunization with different viral vectors expressing protein VP7 of the related BTV conferred homologous and heterologous protection in the IFNAR(−/−) mouse model and natural BTV hosts (51–53). Considering these data, we formulated VP2 or VP7 as target antigens in our viral vector-based vaccine candidates.

In this work, we engineered MVA viral vectors expressing serotype 8 VP2 or serotype 2 VP7 proteins of EHDV as potential immunogenic prototypes. We evaluated the immunogenicity and the homologous protection against EHDV induced by the vaccine candidates in IFNAR(−/−) mice, recently established as a valid animal model for EHDV infection. Finally, we tested the multiserotype and DIVA character of this viral vector vaccine candidates in the IFNAR(−/−) mouse model.

## RESULTS

### Evaluation of EHDV-8 VP2 or EHDV-2 VP7 expression from recombinant MVA

The expression of heterologous antigens downstream of the F13L gene in the MVA genome leads to the induction of potent immune responses in immunized animals (54). Moreover, recent data from our laboratory have shown that cloning of heterologous antigens in the F13L locus of the MVA genome induced a strong immune response against the antigen of interest. Considering this, we generated recombinant MVAs that individually express the gene encoding the EHDV-8 VP2 protein (MVA-VP2) or the EHDV-2 VP7 protein (MVA-VP7) cloned in the F13L locus.

**Fig 1:**
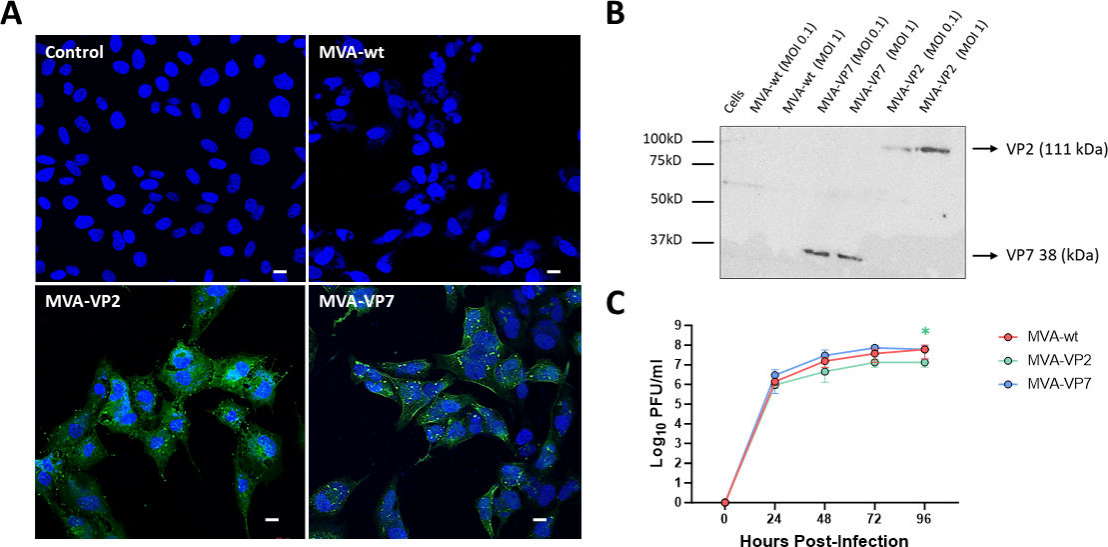
Expression analysis of heterologous EHDV proteins by recombinant MVAs. (**A**) Indirect immunofluorescence of DF-1 cells infected (MOI  =  1) with MVA-wt, MVA-VP2, MVA-VP7, or non-infected (control). VP2 and VP7 proteins (green) were detected using a mouse polyclonal hyperimmune serum against EHDV-8. Nuclei were stained with DAPI. Scale bars 20  µm. (**B**) Immunoblot analysis of non-infected DF-1 cells or infected (MOI = 0.1 and 1) with MVA-wt, MVA-VP2, or MVA-VP7 at 24 hpi using a mouse hyperimmune serum against EHDV-8. Numbers indicate relative molecular mass in kilodaltons (kDa). (**C**) Viral growth kinetics of MVA-wt, MVA-VP2, and MVA-VP7. Monolayers of permissive DF-1 cells were infected with MVA-wt, MVA-VP2, or MVA-VP7 (MOI = 0.1). At 0, 24, 48, 72, and 96 hpi, virus titers in cell lysates were quantified by plaque assay. The assay was performed in triplicate. Points represent the mean log_10_ PFU/mL value of each group. Statistical differences between replication curves of MVA-wt, MVA-VP2, and MVA-VP7 were calculated by multiple t test analysis using Mann–Whitney non-parametric test. * *P* < 0.05.

The correct expression of the heterologous EHDV antigens cloned in the recombinant MVAs was confirmed by immunofluorescence assay (IFA) and immunoblot. DF-1 cells were infected with MVA-VP2, and we observed the expression of this protein throughout the cytoplasm of the infected cells (Fig. 1A). Similarly, DF-1 cells infected with MVA-VP7 showed a cytoplasmic punctuated pattern corresponding to the expression of the heterologous gene. Non-infected or MVA wild-type (MVA-wt) infected cells did not show evidence of a specific signal of VP2 or VP7 in any case. Immunoblotting confirmed the expression of both heterologous EHDV antigens in MVA-infected DF-1 cell lysates (Fig. 1B). Protein bands corresponding to molecular weights of proteins VP2 or VP7 were detected in MVA-VP2 and MVA-VP7 infected cells but not in mock-infected or MVA-wt infected cells.

To analyze whether the expression of EHDV proteins affects MVA replication in cell culture, we studied the growth kinetics of both recombinant MVA in DF-1 cells. No differences were found between the growth kinetics of MVA-wt and MVA-VP7 (Fig. 1C). Despite MVA-VP2 viral titers were very similar to MVA-wt at 24 hpi, slightly lower MVA-VP2 viral titers were observed at 48, 72, and 96 hpi compared with MVA-wt (Fig. 1C), indicating that expression of VP2 mildly weakens MVA vector replication in DF-1 cells.

Altogether, these data confirm the efficient expression of these EHDV proteins cloned in the MVA vaccine vectors to be used for immunization in IFNAR(−/−) mice.

### MVAs expressing VP2 or VP7 protect against a lethal challenge with EHDV-8

**Fig 2:**
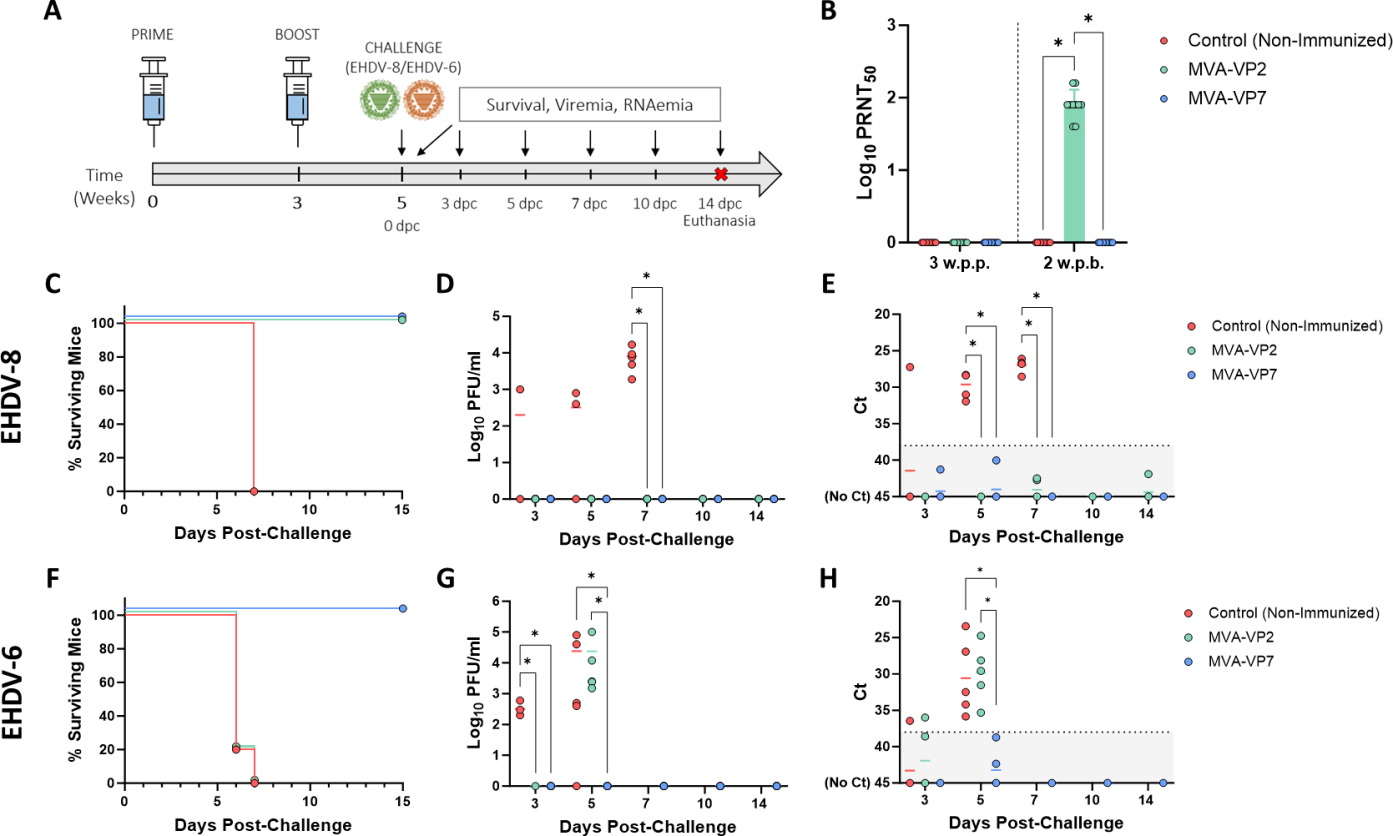
Protection of immunized IFNAR(−/−) mice against a lethal challenge with EHDV-8 or EHDV-6. (**A**) Groups of IFNAR(−/−) mice (*n* = 5) were immunized with two doses of MVA-VP2 or MVA-VP7. Immunized and non-immunized mice were challenged with a lethal dose of EHDV-8 or EHDV-6. In both cases, a group was non-immunized (control). (**B**) Neutralizing antibody titers against EHDV-8 in immunized animals by plaque reduction neutralization assay. Serum was collected from blood samples harvested 3 weeks post-primer and 2 weeks post-boost. Points represent individual neutralizing titer for each mouse, bars represent mean values of each group, and error bars represent SD. Differences between groups were calculated by multiple t test analysis using the Sidak–Bonferroni method. *P < 0.05. Cut-off: 0.69 (log10 5). (**C and F**) Survival rates after challenge. Survival curves were found statistically significant compared with survival curves of control animals as calculated by log-rank test (*P* < 0.05). (**D and G**) Titers of infectious EHDV-6 or EHDV-8 recovered in blood of IFNAR(−/−) mice after viral inoculation calculated by plaque assays on Vero cells. Points represent individual viral titer for each mouse, and lines of the corresponding color represent the mean viral titer of each group. Differences between groups were calculated by multiple t test analysis using the Sidak–Bonferroni method. * *P* < 0.05. (**E and H**) RNAemia analyzed by RT-qPCR after viral inoculation of IFNAR(−/−). Expression of mRNA of segment 9 (encoding VP6 and NS4 proteins) was quantified at 3, 5, 7, 10, and 14 dpc. Results were expressed as Ct (left y axis). The real-time RT-qPCR specific for EHDV segment 9 was performed as described by Maan et al. (59). Points represent individual Ct for each mouse and lines of the corresponding color represent the mean Ct value of each group. Differences between groups were calculated by multiple t test analysis using the Sidak–Bonferroni method. * *P* < 0.05.

The utility of the IFNAR(−/−) mouse model for preclinical vaccine research against BTV and AHSV has been confirmed (55–57), and we recently established this mouse model for vaccine efficacy evaluation against EHDV (58). To test the protective capacity of our recombinant MVAs against EHDV, we intraperitoneally immunized groups of adult IFNAR(−/−) mice (*n* = 5), including two groups, each of them being inoculated with two successive doses (10^7^ PFU) of MVA-VP2 or MVA-VP7, at a 3-week interval. Two additional groups were non-immunized (control). Two weeks after the booster dose, mice were subcutaneously challenged with a lethal dose (100 PFU) of EHDV-8 or EHDV-6 (58) (Fig. 2A). Subsequently, mice were monitored for survival, and viremia and RNAemia were analyzed by plaque assay and RT-qPCR, respectively.

Plaque reduction neutralization tests (PRNT) specific of EHDV-8 were conducted in sera collected at 4 weeks post-prime (wpp) and 2 weeks post-boost (wpb). Virus-neutralizing antibodies were just detected in all mice immunized with two doses of MVA-VP2 (Fig. 2B) but not in mice immunized with two doses of MVA-VP7. The vaccine candidates induced no cross-neutralizing response against EHDV-6 before challenge (data not shown).

After inoculation with a lethal dose of EHDV-8, non-immunized control mice succumbed to EHDV infection by day 7 post-challenge, showing clinical signs characteristics of EHDV infection in IFNAR(−/−) mice (58) (Fig. 2C). Moreover, these control animals displayed high levels of viremia and RNAemia at 5 and 7 days post-challenge (dpc) (Fig. 2D and E). In contrast, groups of animals immunized with either MVA-VP2 or MVA-VP7 and challenged with EHDV-8 showed a 100% survival rate (Fig. 2C), and no infectious virus was detected in blood (Fig. 2D). Moreover, detectable (Ct ≤38) viral RNA levels were not observed after EHDV-8 challenge (Fig. 2E).

To test the multiserotype character of our recombinant vaccine candidates, groups of immunized mice were also challenged with a lethal dose of EHDV-6. Control animals died between days 6 and 7 post-challenge (Fig. 2F). Unsurprisingly, immunization with two doses of MVA-VP2 did not prevent animals from succumbing to EHDV-6 infection. Indeed, these animals died at the same time than those of the control group (Fig. 2F). Besides, viral replication was identical between the non-immunized control group and the MVA-VP2 immunized animals (Fig. 2G and H). In contrast, all MVA-VP7 immunized animals survived to EHDV-6 inoculation (Fig. 2E), and most importantly, none of them showed detectable levels of viraemia or RNAemia after challenge (Fig. 2G and H).

**Fig 3:**
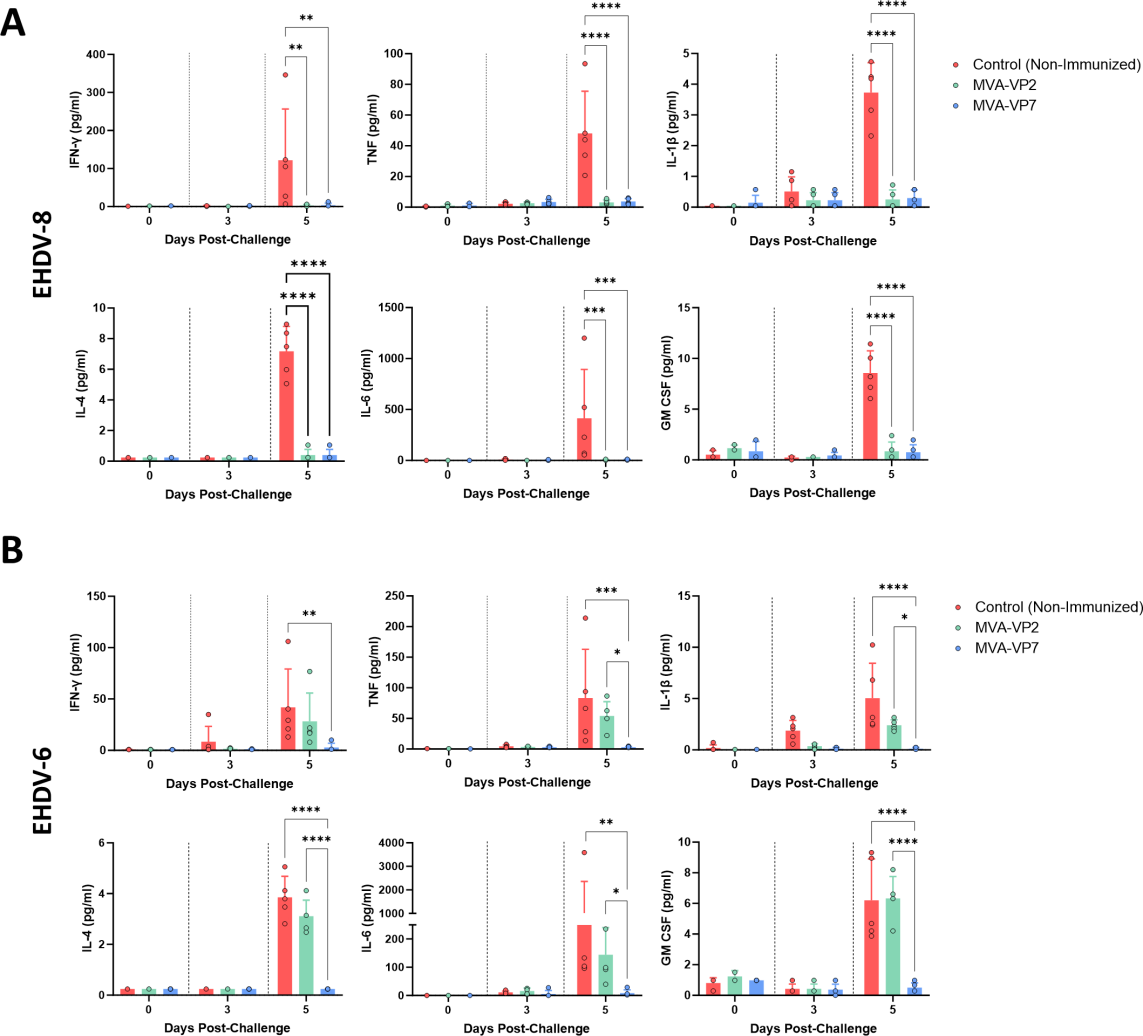
Proinflammatory cytokine changes in IFNAR(−/−) mice challenged with EHDV-8 or EHDV-6. Cytokine production measured in sera from immunized and non-immunized mice after challenge with (**A**) EHDV-8 or (**B**) EHDV-6 by Luminex immunoassays. Points represent individual values for each mouse, bars represent mean values of each group and error bars represent SD. Asterisks denote significant differences between immunized and non-immunized control. *P <  0.05, **P <  0.002, ***P <  0.001, ****P <  0.0001 using two-way ANOVA (*post hoc* Tukey test for multiple comparisons).

An important aspect that significantly contributes to the pathogenesis caused by EHDV infection in natural hosts and IFNAR (−/−) mice is the induction of the so-called cytokine storm (58, 60, 61). To analyze whether our MVA vaccine candidates protect against cytokine storm, we measured circulating levels of proinflammatory cytokines in control and immunized IFNAR(−/−) mice sera after challenge with EHDV-8 or EHDV-6. Non-immunized animals displayed an increase in IFN-γ, TNF, IL-1β, IL-4, IL-6, and GM-CSF after inoculation with EHDV-8 or EHDV-6 at day 5 post-challenge (Fig. 3A and B). After challenge with EHDV-8, mice immunized with MVA-VP2 or MVA-VP7 did not show evidences of this “cytokine storm” as they displayed significantly lower levels of these proinflammatory cytokines compared with the control group at 5 dpc (Fig. 3A). Unsurprisingly, immunization with MVA-VP2 did not block the exacerbated proinflammatory response induced by EHDV-6, with levels of these circulating cytokines comparable to the control group (Fig. 3B). Importantly, we did observe this blockage in mice immunized with MVA-VP7 after challenge with EHDV-6, which displayed significantly lower levels of IFN-γ, TNF, IL-1β, IL-4, IL-6, and GM-CSF compared with the non-immunized and the MVA-VP2 immunized groups (Fig. 3B).

Overall, these data point out the potential of both recombinant vaccine candidates to induce full protection against EHDV-8. Moreover, we also confirmed the capacity of VP7 delivered by a recombinant MVA to confer multiserotype protection in IFNAR(−/−) mice.

### Detection of antibodies specific of VP2 and VP7 of EHDV in sera

**Fig 4:**
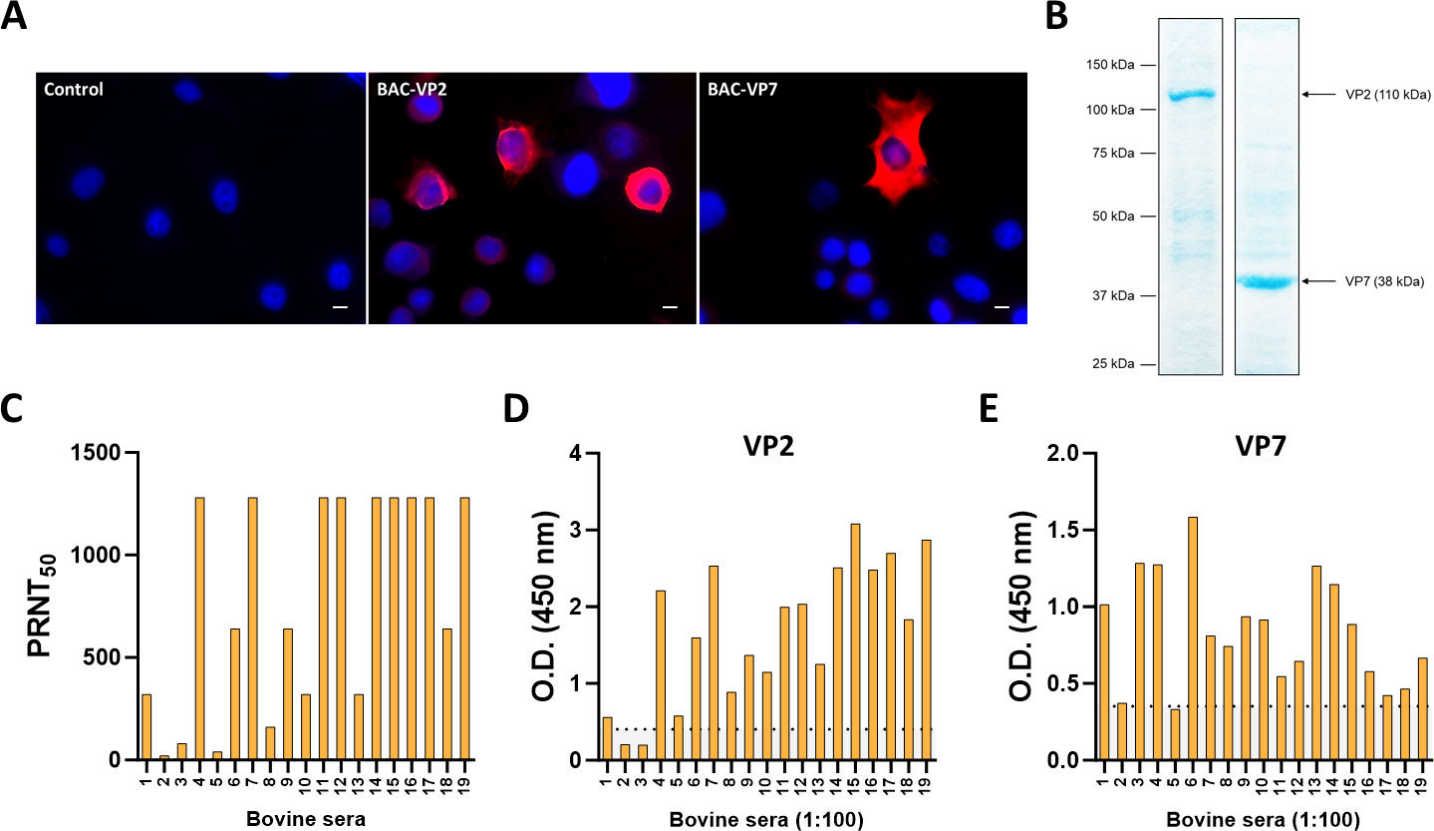
Expression of EHDV VP2 and VP7 proteins in the baculovirus system for detection of EHDV specific antibodies. (**A**) Indirect immunofluorescence of H5 cells infected (MOI  =  1) with BAC-VP2, BAC-VP7, or non-infected (control). VP2 and VP7 proteins (red) were detected using a mouse polyclonal hyperimmune serum against EHDV-6. Nuclei were stained with DAPI. Scale bars 20  µm. (**B**) SDS acrylamide gels 12% of purified proteins VP2 of EHDV-8 and VP7 of EHDV-2 dyed with coomassie blue. Numbers indicate relative molecular mass in kDa. (**C**) Neutralizing antibody titers against EHDV-8 in cattle sera by plaque reduction neutralization assay. (**D and E**) Field bovine sera were analyzed by ELISA for the presence of IgG immunoglobulins against (**D**) VP2 and (**E**) VP7 proteins. Bars represent individual values for each animal. Dotted lines represent the cut-off established as indicated in Materials and Methods.

To analyze the adaptive immune responses elicited by the vaccine candidates generated in this work, the recombinant proteins VP2 of EHDV-8 and VP7 of EHDV-2 were expressed by using the baculovirus (BAC) expression system. H5 cells were infected with BAC-VP2 or BAC-VP7, and the EHDV protein expression was analyzed by indirect immunofluorescence assay (IFA) at 24 h post-infection (hpi) by using a mouse polyclonal hyperimmune serum against EHDV-6. Whereas no fluorescent signal was detected in non-infected H5 cells, specific signals of proteins VP2 and VP7 were observed in H5 cells infected with BAC-VP2 or BAC-VP7, respectively (Fig. 4A).

Proteins were purified from H5 cells infected with BAC-VP2 or BAC-VP7, as described in Materials and Methods (Fig. 4B). Thereafter, we wondered whether cattle sera could recognize these EHDV proteins. To do so, we used a set of unknown bovine field sera collected during 2023 in regions where EHDV-8 circulated, gently handed by Laboratorio Central de Veterinaria (LCV, MAPA). First, we determined PRNT_50_ titers in these sera samples against EHDV-8 (Fig. 4C). In total, 17 bovine samples (90%) showed neutralizing activity by PRNT_50_ and also detected recombinant proteins VP2 and VP7 of EHDV by indirect ELISA (Fig. 4D and E). Unsurprisingly, optical densities (ODs) obtained against protein VP2 of EHDV-8 correlated with PRNT_50_ values against EHDV-8 in cattle (Spearman r = 0.9254; *P* < 0.0001). This correlation was not observed for protein VP7. These data confirm that the baculovirus-expressed proteins VP2 of EHDV-8 and VP7 of EHDV-2 can be applied for detection of VP2 or VP7 specific antibody responses and could provide a first approach to study the DIVA properties of our novel recombinant vaccine candidates.

**Fig 5:**
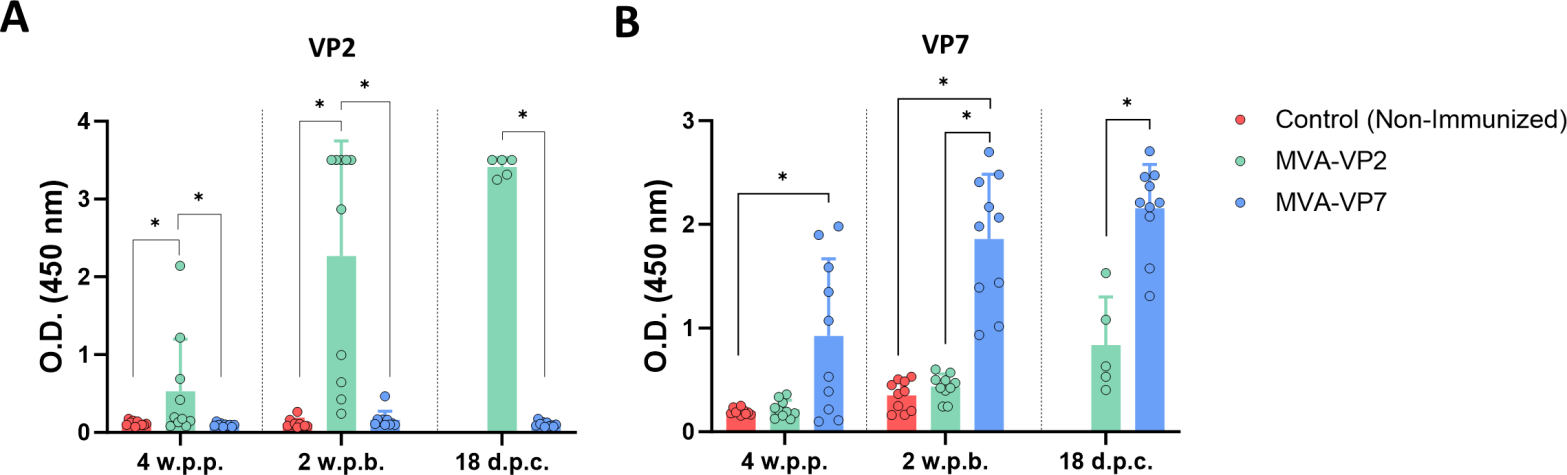
Analysis of the presence of antibodies specific of VP2 and VP7 in serum of immunized IFNAR(−/−) mice by ELISA. Sera of immunized and non-immunized IFNAR(−/−) mice were analyzed by ELISA for the presence of antibodies against (**A**) VP2 and (**B**) VP7 proteins. Serum of immunized mice was collected 4 weeks after the primer, 2 weeks post-boost, and 18 days post-challenge with EHDV-8 or EHDV-6 and were analyzed by ELISA as described in Materials and Methods. Points represent individual values for each mouse, bars represent the mean values of each group, and error bars represent SD. Asterisks denote significant differences between groups (*P*   <  0.05) (Mann–Whitney U test). *P <  0.05.

Subsequently, we used these recombinant EHDV proteins to study the DIVA potential of our MVA-based vaccine candidates. Sera from non-immunized and MVA-immunized IFNAR(−/−) mice were analyzed for their reactivity to VP2 and VP7 proteins by indirect ELISA. After 4 weeks post-prime, some mice immunized with MVA-VP2 displayed low antibody responses against protein VP2 (Fig. 5A). Similarly, immunization with one dose of MVA-VP7 induced low titers of anti-VP7 antibodies in some immunized animals (Fig. 5B). After the booster dose, a rise in antibody levels against VP2 or VP7 was observed in sera from IFNAR(−/−) mice immunized with MVA-VP2 or MVA-VP7, respectively (Fig. 5A and B). Importantly, MVA-VP2 immunized animals did not develop an antibody response against protein VP7 and MVA-VP7 immnuzed mice against protein VP2 (Fig. 5A and B) which suggests that both vaccine candidates possess DIVA character.

Moreover, antibody response against VP2 did not arise after challenge in those groups of MVA-VP7 immunized animals that survived to lethal challenge with EHDV-8 or EHDV-6 (Fig. 5A). Similarly, some mice immunized with MVA-VP2 did not show an antibody response to VP7 after challenge with EHDV-8 (Fig. 5B), which confirmed the abrogation of viral replication induced by the vaccine candidates.

### Multiserotype protection induced by MVA-VP7 is mediated by a cellular immune response

The core protein VP7 is one of the most conserved proteins among EHDV serotypes, with a percentage of identity of the amino acid sequences among EHDV serotypes higher than 90% (49, 50). Indeed, we identified theoretical CD8+ T-cell epitopes within the sequence of protein VP7 using the prediction algorithms IEDB and SYFPEITHI and considering the major histocompatibility complex (MHC) haplotype from the mice used in the study (Table 1).

**TABLE 1:** VP7 peptides identified from the epitope prediction in H-2 Db haplotype

Database	Initial position	Length	Sequence	Score
SYFPEITHY (Database of MHC Ligands and Peptide Motifs)	283	9	AAILNRTTL	31
	83	9	DYIQNLATI	27
	72	9	AANLNVGNI	25
	290	9	TLPNNIPPI	24
	324	9	APDFNLFGI	24
	175	9	FQNQNDPIM	23
	24	9	VVESNVLEI	22
	302	9	NDRENVLLL	22
	37	9	INRYNGLTL	21
	55	9	QEQRNEMFF	20
IEDB (Immune Epitope Database and Analysis Resource)	283	9	AAILNRTTL	0.99423
	175	9	FQNQNDPIM	0.632035
	290	9	TLPNNIPPI	0.619419
	72	9	AANLNVGNI	0.600032
	144	9	RAGQNITTA	0.59125
	95	9	ATPEIPYTM	0.497133
	101	9	YTMESANEI	0.403732
	341	9	RAVAQNAYM	0.375087
	314	9	SALADAFSV	0.355126
	77	9	VGNISPDYI	0.294326

In the light of the above, we decided to evaluate the cellular immune response elicited by the VP7 EHDV protein when delivered by the MVA viral vaccine vector in IFNAR(−/−) mice. For this purpose, a group of IFNAR(−/−) mice (*n* = 4) was immunized with two doses (10^7^ PFU) of MVA-VP7 in a 4-week interval. A group of mice was non-immunized (control). Two weeks post-boost, the animals were sacrificed, and spleens were collected. Splenic cellular immune responses were measured by IFN-γ ELISpot and intracellular cytokine staining (ICS).

**Fig 6:**
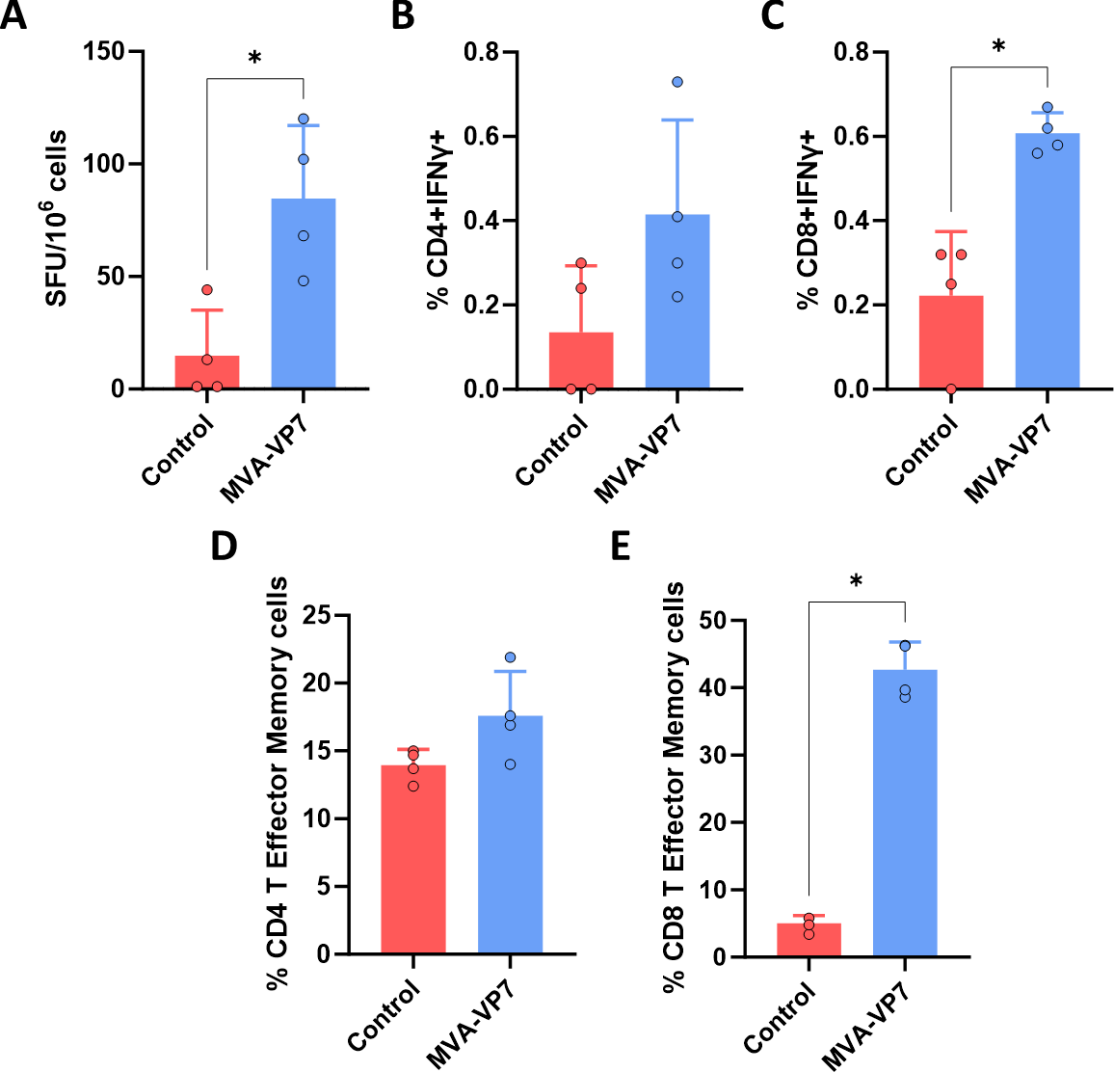
Cellular immune responses against EHDV in MVA-VP7-immunized mice. A group of IFNAR(−/−) mice (*n* = 5) was immunized with two doses of MVA-VP7. A group of mice was left untreated. Two weeks post-boost, the animals were sacrificed, and their spleens were harvested for the analysis of the T cell response. (**A**) ELISpot assay measuring IFN-γ-secreting cells in the spleen of immunized mice. (**B and C**) Percentage of CD4+ IFN-γ+ T cells (**B**) and CD8+ IFN-γ+ T cells (**C**) after stimulation with VP7. (**D and E**) Percentage of CD4+ T effector memory (TEM) (**D**) and CD8+ TEM cells (**E**). CD44 and CD62L expression was used to identify TEM subpopulations. Points represent individual values for each mouse, bars represent the mean values of each group, and error bars represent SD. Asterisks denote significant differences between immunized and control mice (*P* < 0.05) (Mann–Whitney U test). *P < 0.05.

After restimulation with purified protein VP7, analysis of murine splenocyte responses by IFN-γ ELISpot assay showed the induction of a statistically significant EHDV VP7 protein-specific cellular immune response in MVA-VP7-vaccinated animals (Fig. 6A). We also measured IFN-γ production in CD4+ and CD8+ T cells by ICS after restimulation of splenocytes from immunized and non-immunized mice with the purified recombinant protein VP7. After restimulation, the MVA-VP7-immunized animals showed a non-significant increase in the percentage of CD4+ IFN-γ+ cells in comparison with the non-immunized group (Fig. 6B). More importantly, splenocytes of mice immunized with MVA-VP7 displayed significant levels of CD8+ IFN-γ+ (Fig. 6C) upon restimulation with VP7 recombinant protein. We also determined the percentage of the T effector memory (TEM) EHDV-specific CD4+ and CD8+ cells by evaluating the presence of CD44 and CD62L surface markers. Meanwhile, no significant differences were observed in the percentage of TEM CD4+ cells between the MVA-VP7 and the non-immunized group (Fig. 6D), restimulation with protein VP7 led to a pronounced TEM CD8+ response (Fig. 6E). Overall, these data reflect that the MVA-VP7 induces a cytotoxic CD8+ T-cell memory response.

## DISCUSSION

The recent incursion and subsequent propagation of EHDV-8 in Europe have outlined the need for countermeasures against this viral disease. The success of vaccination campaigns against the highly related BTV is beyond doubt. Therefore, controlling EHDV through vaccination is potentially achievable. However, vaccine research against EHDV has been negligible. Besides, although inactivated and LAVs vaccines can be effective, their lack of broad protective immunity against multiple EHDV serotypes, the impossibility to apply a DIVA strategy or safety concerns magnify the need for development of recombinant vaccines. Thus, to the best of our knowledge, we developed the first recombinant DIVA vaccine candidate against EHDV-8 and the first multiserotype vaccine candidate against EHDV.

Protein VP2 has been the unique antigen included in the evaluated subunit recombinant vaccine against EHDV (40). As VP2 is the inductor of neutralizing responses, and it is a highly variable protein among EHDV serotypes (19), protection induced by VP2 is serotype-specific. In this sense, current vaccines against EHDV cannot be applied for control of EHDV-8. Recently, Spedicato et al. have evaluated an inactivated vaccine against EHDV-8 (62). As aforementioned, inactivated vaccines imply some disadvantages that emphasize the need for the development of recombinant vaccines. Here, in a part of this work, we present a recombinant viral vector MVA vaccine based on the expression of protein VP2 of EHDV-8. This vaccine candidate afforded complete protection against the homologous EHDV-8 in the IFNAR(−/−) mouse model, avoiding viral replication and the progression of disease. Despite promising results, correlation between protective responses in the IFNAR(−/−) mouse model and natural hosts of EHDV needs to be ascertained (58). Nonetheless, several works have pointed out that results on immunogenicity and protection induced by a wide range of recombinant vaccines against BTV correlates between IFNAR(−/−) mice and natural hosts of this viral disease (53, 55, 56, 63, 64). Moreover, an inactivated vaccine against EHDV-8 was proven immunogenic and protective against the virus in the IFNAR(−/−) mouse model, and recently, another inactivated EHDV-8 vaccine also showed efficacy in natural hosts of EHDV (58, 62). Therefore, it is quite probable that the immunogenic and protective capacity of this MVA-VP2 is also maintained in natural hosts, although further work will be required to confirm this.

Unsurprisingly, protection induced by the EHDV-8 inactivated vaccine or the MVA-VP2 relied on homologous neutralizing responses (58, 62), which is incompatible with multiserotype protection against heterologous EHDV serotypes. As it is one of the major drawbacks of orbivirus vaccines, other antigens highly conserved through EHDV serotypes should be targeted. Therefore, we evaluated the protein VP7, almost identical among EHDV serotypes, as a potential multiserotype antigen. Interestingly, immunization with MVA-VP7 conferred complete protection against EHDV-8 in IFNAR(−/−) mice, and no differences were observed compared with the protection induced by MVA-VP2. The gene encoding for VP7 cloned in the MVA came from EHDV-2, so that protection conferred against EHDV-8 already indicated the multiserotype potential of this vaccine candidate. In any case, the high degree of protection against EHDV-6 elicited by MVA-VP7 confirmed its broad cross-protective immunogenicity. For BTV, expression of the core protein VP7 by recombinant MVA or canine adenoviral type two vectors did not induce a significant degree of protection in immunized animals, being immunogenic but not fully protective against BTV, as viral replication and disease progression were not blocked (65, 66). However, expression of protein VP7 by other viral vectors led to homologous and heterologous protection in the IFNAR(−/−) mouse model and natural BTV hosts (51–53). In this sense, the protective role of the BTV antigen VP7 needs to be ascertained, although differences between EHDV and BTV could exist regarding the antigenicity of their viral proteins. The identification of antigens able to confer multiserotype protective responses against EHDV is key due to the uncertain epidemiology of this disease. The arrival of novel serotypes and strains of EHDV to non-endemic regions is frequent (67–69). In this line, current circulation of EHDV-6 in North Africa implies a high risk of distribution to non-endemic European regions as it occurred with EHDV-8 (70). Therefore, future vaccine design should consider the formulation of proteins with high sequence homology between EHDV serotypes, such as VP7, in vaccine composition. Besides, other conserved antigens should be explored, as it was the case of proteins NS1 or NS2 of BTV (56, 64, 71).

For BTV, protection induced by expression of protein VP7 is mainly mediated by a potent cytotoxic T cell response (51–53). Here, we observed that protection conferred by MVA-VP7 occurred in absence of NAbs against EHDV. Besides, we identified the presence of theoretical CD8+ T epitopes within the sequence of protein VP7. Considering this, we confirmed by ELISpot and ICS the potential of the protein VP7 of EHDV delivered by MVA to elicit a specific cytotoxic T cell response, which seems to be responsible for the high degree of multiserotype protection observed in IFNAR(−/−) mice. However, the immunogenicity as well as the protective efficacy must be confirmed in natural hosts of EHDV, as the adaptive response might differ between mice and other mammals (72–74). In any case, considering that NAb titers do not always correlate with protection against BTV (75, 76), combination of both arms of the adaptive immune response, virus NAbs and cytotoxic T lymphocytes, is crucial for the development of a long lasting and protective immunity against BTV (77, 78) and should be the case for EHDV. Therefore, prospects on vaccine formulation against EHDV should consider the combination of protein VP2 and VP7, which might reinforce both homologous and heterologous protective responses.

As elimination of nonstructural EHDV proteins is highly difficult during the purification process (39), EHDV-inactivated vaccines cannot permit to distinguish between vaccinated and naturally infected animals by serological assays (62, 79). For this reason, the development of recombinant vaccines is needed to provide DIVA vaccine approaches against EHDV, which will allow a correct viral surveillance in the field. An important advantage of the recombinant EHDV vaccine candidates generated in this work is that they express one single EHDV antigen, which make them potential marker vaccines with DIVA properties. In this work, we expressed and purified proteins VP2 and VP7 of EHDV to provide a first insight into the likely DIVA aspect of our recombinant vaccines. Our results show the induction of an antibody response against VP2 after immunization with the MVA expressing this EHDV antigen, but not for VP7, which was not included in the recombinant vaccine. Similarly, immunization with MVA-VP7 induced antibodies specific of protein VP7 but not of protein VP2. Therefore, these results suggest the DIVA potential of these MVA-based vaccine candidates. However, it is important to note that the evaluation of the DIVA character presented in this work is highly limited by the indirect ELISA assays performed here as we could only test a low number of cattle sera, and most of them showed a neutralizing response to EHDV-8. The difficulties to obtain a significant number of known positive and negative sera samples of EHDV as well as the predominance of positive samples hindered the study of the sensitivity/specificity of the ELISA assays. In consequence, despite the design of the recombinant vaccine and our data suggest the DIVA character of the recombinant MVA vaccine, it should be further confirmed.

Several approaches have been implemented for next-generation vaccine design against orbiviruses, including Virus-Like particles (VLPs) or optimized LAVs (DISA and DISC vaccines) (41, 80, 81). VLPs provide robust protection through a strong induction of neutralizing humoral responses, being a weak stimulus for the cell-mediated arm of the adaptive immune response (82, 83). The usefulness of viral vectors relies on their capacity to infect and transiently express the antigen of interest in host cells, which leads to potent humoral and cellular immune responses (45). The unique novel orbivirus-vaccine approaches comparable to viral vector vaccines are DISA and DISC vaccines. These LAVs can infect host cells, express orbivirus antigens, but their replication is abortive as for MVA. Nonetheless, the protection induced by DISC and DISA vaccines is still mainly mediated by serotype-specific NAbs, although expression of other conserved antigens that mediate multiserotype protection occurs (84, 85). Therefore, a cocktail of DISA and DISC viruses of different serotypes is needed to achieve multiserotype protection as for VLPs (86–88). In contrast, we present a single recombinant MVA-based vaccine that stimulates a cellular immune response, conferring multiserotype protection against EHDV. Further, the immunogenicity of this multiserotype vaccine candidate could be optimized by cloning of protein VP2 of EHDV-8 in another locus of the MVA expressing VP7, combining both arms of the adaptive immune response as for BTV (65).

In summary, this study presents safe and adjuvant-free MVA-based vaccine candidates likely compatible with a DIVA strategy. Moreover, we identified the protein VP7 as an applicable antigen for potential vaccines against EHDV able to induce a multiserotype protection mediated by a potent EHDV CD8+ T-cell immune response, which addresses one of the major challenges in vaccination against EHDV.

## MATERIALS AND METHODS

### Cell lines and viruses

Chicken embryo fibroblasts (DF-1) (ATCC, Cat. No. CRL-12203) were grown in Dulbecco’s Modified Eagle’s medium (DMEM) (Biowest, Nuaillé, France) supplemented with 2  mM glutamine (Gibco, Waltham, MA, USA) and 10% fetal calf serum (FCS) (Gibco, Waltham, MA, USA). Green monkey kidney cells (Vero) (ATCC, Cat. No. CCL-81) and BHK-21 cells (ATCC; catalog no. CCL-10) were grown in Dulbecco’s Modified Eagle’s medium (DMEM) (Biowest, Nuaillé, France) supplemented with 2 mM glutamine (Gibco, Waltham, MA, USA) and 5% FCS (Gibco, Waltham, MA, USA). Insect cells High Five (H5) (Thermo Fisher Scientific, NY, USA) were grown in TC-100 medium supplemented with 10% fetal calf serum (FCS).

EHDV serotype 2 (EHDV-2) (CAN 1962/01), EHDV serotype 6 (EHDV-6) (EHDV-6 MOR2006/07) and EHDV serotype 8 (EHDV-8/Spa) (isolated in Spain, 2022) were used in the experiments. EHDV-8 Spanish isolate was isolated from cattle blood in KC insect cells and passaged twice in BHK cells. Viruses were passaged once in KC insect cells, and virus-working stocks were grown in BHK cells. Virus stocks and titrations were performed by standard methods previously described (79).

### Generation of recombinant MVA vaccine vectors

The procedure to generate MVA viral vectors has been depicted elsewhere (89). We generated recombinant MVAs containing genes encoding for EHDV-8 VP2 (Accession number: OQ625430.1) or EHDV-2 VP7 (Accession number: AM745003.1) proteins placed in the F13L locus. For this purpose, transfer plasmids pMVA containing genes encoding for VP2 from EHDV-8 (EHDV-8/Spa) or VP7 from EHDV-2 (CAN 1962/01) were constructed. Shortly, cDNAs corresponding to segments 2 and 7 encoding for VP2 or VP7 protein were generated from total RNA of EHDV-2 or EHDV-8 infected cells, respectively by using SuperScript™ III Reverse Transcriptase (Thermo Fisher Scientific, NY, USA), following manufacturer’s instruction. Subsequently, segments 2 and 7 were amplified with primers specified in Table 2. The restriction sites EcoRI and BamHI were introduced at the 5′ and 3′ ends of the PCR products. The DNA inserts were digested with EcoRI and BamHI restriction enzymes, and the DNA inserts were cloned into the MVA transfer plasmid pMVA-β-Gus (90) previously digested with the same restriction enzymes. Subsequently, plasmids pMVA-VP2 or pMVA-VP7 were transfected in DF-1 cells infected with MVAΔF13L that encodes dsRed marker instead of the native F13L ORF at a multiplicity of infection (MOI) of 1 using Lipofectamine™ 3000 Transfection Reagent (Invitrogen™, CA, USA), following the protocol facilitated by the manufacturer. Cell cultures were harvested at 48 h post-infection (hpi), and rMVAs were purified by plaque-picking and fluorescent selection in a Zeiss Axio fluorescence microscope (Zeiss, Oberkochen, Germany). Complementary, rMVAs were cloned at least five times by plaque assay for a greater purification.

**TABLE 2:** Primers designed to generate recombinant MVAs and recombinant BAC

Primer	Sequence
Fw-EcoRI-VP2^*a*^^,*b*^	5′-ATgaattcATGGATTCTGTAGAATTTGCTATCC-3′
RS-BamHI-VP2^*a*^	5′-ATggatccTCAGTTCATCAATTTTGTTACCAACTCGTGG-3′
Fw-EcoRI-VP7^*a*^^,*b*^	5′-ATgaattcATGGACACGATTGCAGCAAGAGCTC-3′
RS-BamHI-VP7^*a*^	5′-ATggatccTCACACATAGGCGTTTTGAGC-3′
Rs-NotI-VP2^*b*^	5′-ATgcggccgcTCAGTTCATCAATTTTGTTACCAACTCGTGG-3′
Rs-NotI-VP7^*b*^	5′-ATgcggccgcTCACACATAGGCGTTTTGAGC-3′

^*a*^ Primers designed to generate pMVA-VP2 or pMVA-VP7. EcoRI and BamHI restriction sites are represented by lowercase letters.

^*b*^ Primers designed to generate HTA-VP2 or HTA-VP7. EcoRI and NotI restriction sites are represented by lowercase letters.

### Generation of recombinant baculovirus expressing VP2 or VP7

To obtain recombinant proteins VP2 of EHDV-8 and VP7 of EHDV-2, recombinant baculoviruses (rBAC) BAC-VP2 and BAC-VP7 were generated using the Bac-to-Bac system (Invitrogen, Barcelona, Spain), following the supplier protocols. Briefly, plasmid pMVA-VP2 or pMVA-VP7 containing the sequence coding for VP2 protein of EHDV serotype 8 or VP7 protein of EHDV-2 was used to amplify the sequences of VP2 or VP7 with specific primers (Table 2). The PCR products were then cloned into plasmid pFastBac HTA (Invitrogen, Barcelona, Spain) digested with EcoRI and NotI to obtain the transfer vectors HTA-VP2 and HTA-VP7 that were used to generate the recombinant baculoviruses that express the VP2 or VP7 EHDV proteins in insect H5 cells, named BAC-VP2 and BAC-VP7. The EHDV proteins with a N-terminal 6× Histidine tag were purified using the ProBond Purification System (Invitrogen, Barcelona, Spain) following the procedure indicated by the manufacturer for protein purification under native conditions. Purified proteins were resolved in a 12% SDS-PAGE and stained with PageBlue Protein Staining Solution (Thermo Fisher Scientific, MD, USA).

### Indirect immunofluorescence microscopy

DF-1 cells were grown in glass coverslips and infected with MVA-wt, MVA-VP2, or MVA-VP7 at a MOI of 1, or non-infected. H5 cells were DF-1 cells were grown in glass coverslips and infected with BAC-VP2 or BAC-VP7 at a MOI of 1, or non-infected. Twenty-four hours after infection, cell monolayers were fixed for 15 minutes with 4% paraformaldehyde. Fixed cells were blocked with 20% FBS-PBS-Saponine 0.2% (20% blocking solution) for 60 min at room temperature (RT). DF-1 cells were incubated overnight at 4°C with a mouse hyperimmune serum against EHDV-8 [obtained from an *in vivo* infection (58)] (1:500) diluted in PBS-FBS 20%. H5 cells were incubated overnight at 4°C with a mouse hyperimmune serum against EHDV-6 [obtained from an *in vivo* infection (58)] (1:500) diluted in PBS-FBS 20%. After three serial washing steps with PBS, DF-1 cells were incubated for 30 min at room temperature (RT) with Alexa Fluor 488 goat conjugated anti-mouse IgG (Invitrogen™, German Town, MD, USA) (1:1000). H5 cells were incubated for 30 min at room temperature (RT) with Alexa Fluor 594 goat conjugated anti-mouse IgG (Invitrogen™, German Town, MD, USA) (1:1000). Coverslips with infected DF-1 or H5 cells were washed three times with PBS and once with PBS-DAPI (1:10,000), and visualized in a Zeiss Axio fluorescence microscope (Zeiss, Oberkochen, Germany). Adobe Photoshop CS5 Extended (Adobe Systems, CA, USA) was used afterwards for image editing.

### Western blot analysis

DF-1 cells were mock infected or infected with MVA-wt, MVA-VP2 or MVA-VP7 (MOI = 0.1 and 1). At 24 hpi, cells were harvested, washed in PBS containing protease inhibitor cocktail (Sigma-Aldrich, St. Louis, MO, USA), and lysed with RIPA Buffer (Santa Cruz Biotechnology, Dallas, TX, USA). Then, extracts were sonicated for 2 min, and proteins were resolved in 12% SDS-PAGE and blotted onto a nitrocellulose membrane. After a blocking step with 5% low-fat dry milk in TBS Tween-20 (TBST) (blocking buffer), a mouse hyperimmune serum against EHDV-8 [obtained from an *in vivo* infection (58)] (1:500) was applied to membranes of infected DF-1 cells in TBST-Milk 5% and incubated overnight at 4°C. Bound antibody was detected with horseradish peroxidase-conjugated rabbit anti-mouse antibody (Sigma-Aldrich, San Louis, MO, USA) diluted in TBST-Milk 5% (1:10,000) and the ECL detection system (AmershamTM Pharmacia Biotech, Buckinghamshire, UK).

### Analysis of recombinant MVA virus growth

Monolayers of permissive DF-1 cells were infected with MVA-wt, MVA-VP2, or MVA-VP7 at a MOI of 0.1. At 0, 24, 48, and 72 hpi, cells were harvested, and virus titers in cell lysates were determined by a plaque assay in DF-1 cells, as previously described (89).

### Mice

Male and female type I interferon receptor defective mice [IFNAR(−/−)] on A129 Sv/Ev background and A129 mice were used throughout. All mice were matched for age (8 weeks). Mice were housed under pathogen-free conditions and allowed to acclimatize to the biosafety level 3 (BSL3) animal facilities at the Animal Health Research Center (CISA-INIA, CSIC), Madrid, before use.

### Mouse immunization and challenge

Groups of IFNAR(−/−) mice (*n* = 5) were intraperitoneally immunized following a homologous prime–boost regime consisting of two doses of 1 × 10^7^ PFU per mouse of MVA-VP2 or MVA-VP7, administered 3 weeks apart. Two groups of mice (*n* = 5) were left untreated (control). Animals were subcutaneously challenged with a lethal dose of EHDV-8 (100 PFU) or EHDV-6 (100 PFU) 2 weeks post-boost. Sera of immunized and control animals were collected at 3 weeks post-prime and 2 weeks post-boost for the analysis of the humoral immune response. After virus challenge, mice were daily examined for survival and clinical signs, and submandibular blood samples were collected at 3, 5, 7, 10, and 14 dpc for the analysis of viremia and RNAemia by plaque assay in Vero cells and RT-qPCR, respectively. Sera of immunized and control animals were collected at 0, 3 and 5 dpc for the analysis of circulating proinflammatory cytokines.

### Viremia and RNAemia analyses by plaque assay and RT-qPCR

Blood samples were collected from the submandibular plexus of mice with EDTA as anti-coagulant. For the analysis of viremia by plaque assay, 50 µL of blood was diluted in PBS 1× and centrifuged at 3,000 rpm for 10 min. Thereafter, supernatant was removed, and pellet was lysed in 450 µL of sterile water for 2 min. Cell lysis was stopped by adding 50 µL of PBS 10×. Then, samples were inoculated into 12-well plates containing semi-confluent monolayers of Vero cells. Following incubation for 1 h, an agar overlay (DMEM-10%-FBS-0.4%-Noble Agar, Becton Dickinson, MD, USA) was added, and plates were incubated for 5 days at 37°C in 5% CO_2_. Plaques were fixed with 10% formaldehyde and visualized with 2% crystal violet-PBS.

For the analysis of RNAemia by RT-qPCR, RNA was extracted from 50 µL of blood using TRIzol Reagent (Sigma Aldrich, St. Louis, MO, USA) following the protocol established by the manufacturer. RNAemia was analyzed in duplicate by real-time RT-qPCR specific for EHDV segment 9 (encoding for VP6 and NS4). The real-time RT-qPCR specific for EHDV segment 9 was performed using primers and probe described by Maan et al. (59). Only Ct values lower than 38 were considered indicative of viremia (positive) and “No Ct” values were considered as a Ct of 45.

### Determination of circulating levels of cytokines

Sera from immunized and non-immunized mice were collected at 0, 3, and 5 dpc. Circulating cytokine levels were analyzed using a multiplex fluorescent bead immunoassay for quantitative detection of mouse cytokines (ProcartaPlexMouse TH1/Th2 Cytokine Panel 11plex, Invitrogen™, CA, USA). Samples were analyzed with a MAGPIX system (Luminex Corporation, Austin, TX, USA).

### Detection of antibodies specific of VP2 and VP7 by ELISA

MaxiSorp plates (Nunc) (Thermo Fisher Scientific, NY, USA) were coated with EHDV-8 VP2 (100 ng/well) or EHDV-2 VP7 (100 ng/well) purified baculovirus expressed proteins in PBS and incubated overnight at 4°C. Plates were saturated with blocking buffer (PBS-0.05%-Tween 20-5% skim milk). Individual mice or bovine sera diluted in blocking buffer (1:100) were added and incubated for 2 h at 37°C. After three washes in PBS-0.05% Tween 20, plates were incubated for 1 h at 37°C with an anti-mouse-HRP secondary antibody (Bio-Rad, Hercules, CA, USA) (1:2,000) or with an anti-bovine-HRP secondary antibody (Sigma-Aldrich, San Louis, MO, USA) (1:2,000) in blocking buffer. Finally, after three washes in PBS-0.05% Tween 20, the reaction was developed with 50 µL of TMB (Thermo Fisher Scientific, MD, USA) and stopped by adding 50 µL of 3 N H_2_SO_4_ (Merck, Darmstadt, Germany). Results were expressed as optical densities (ODs) measured at 450  nm. The cut-off for bovine ELISA assays was determined as the mean OD + three times the standard deviation of a collection of known negative bovine serum samples.

### Plaque reduction neutralization test

Two-fold dilutions (from 1:5) of heat inactivated mice or bovine sera (56°C for 30 min) were incubated with 100 PFU of EHDV-6 or EHDV-8 for 1 h at 37°C. Two-fold dilutions (from 1:5) of heat-inactivated bovine sera (56°C for 30 min) were incubated with 100 PFU of EHDV-8 for 1 h at 37°C. Then, samples were inoculated into 12-well plates containing semi-confluent monolayers of Vero cells. Following incubation for 1 h, an agar overlay (DMEM-10%-FBS-0.4%-Noble Agar, Becton Dickinson, MD, USA) was added, and plates were incubated for 5 days at 37°C in 5% CO_2_. Plaques were fixed with 10% formaldehyde and visualized with 2% crystal violet-PBS. A 50% plaque reduction neutralization test (PRNT_50_) titer was calculated as the reciprocal (log_10_) of the highest dilution of serum that neutralized 50% of the control virus input. The cut-off is 0.69, log_10_ of the reciprocal of the first dilution 1:5.

### *In silico* VP7 T-CD8 epitope prediction

Amino acid sequence for the non-structural VP7 protein (NCBI accession number: CAN89101) was analyzed using two prediction algorithms available on the web: SYFPEITHI (http://www.syfpeithi.de/) and Immune Epitope DataBase (IEDB Analysis Resource) (www.iedb.org) for the H-2-Db MHC class I for 129/Sv mice to identify theoretical T-CD8 epitopes that could be good binders to H-2-Db MHC.

### *Ex vivo* IFN-γ ELISpot

Groups of IFNAR(−/−) mice (*n* = 4) were nonimmunized or immunized following a homologous prime–boost regimen with MVA-VP7 (10^7^ PFU per dose) in a 3-week interval. A group of mice (*n* = 4) was left untreated (control). All animals were sacrificed at 15  days post-boost, and their spleens were harvested for IFN-γ-ELISPOT analysis.

ELISpot plates containing Immobilon-P membrane (Millipore) were permeabilized with 35% ethanol in sterile water, washed twice with PBS, and coated with 50 µg of the capture antibody, mouse anti-IFN-γ AN18 (BD Pharmingen) at 2.5 µg/mL in PBS, and incubated overnight at 4°C. The plate was blocked with complete medium for 1 h at 37°C, and splenocytes were seeded at a concentration of 2 × 10^5^ cells per well. Cells were stimulated in the presence of 5 µg/mL baculovirus-expressed recombinant VP7 for 18 h at 37°C and 5% CO_2_. An irrelevant protein was used as a negative control, and Phorbol 12-myristate 13-acetate phytohemagglutinin (PHA) (Sigma) at a final concentration of 5 µg/mL was used as a positive control. After 18 h, the plates were washed with distilled water to lyse the cells and five times with PBS. Then, 50 µL per well of biotin-conjugated anti-IFN-γ Ab (BD Pharmingen) diluted 1:100 in PBS was added. Incubation was carried out at room temperature for 2 h in the dark. Then 50 µL of streptavidin–HRP conjugate (BD Biosciences) diluted 1:1,000 in PBS was added. After 1 h of incubation, 50 µL of the TMB developing solution (Mabtech) was added to each well until the spots were detectable, subsequently washing with H_2_O. Once the membranes were dried, the number of spots in each well was determined using an AID iSpot Reader System (Autoimmun Diagnostika, Strassberg, Germany).

### *Ex vivo* flow cytometric analysis

A total of 10^6^ splenocytes per well were stimulated for 18 h with 5  µg/mL of EHDV-2 VP7 protein, concanavalin A (ConA) as a nonspecific stimulus (4  µg/mL) or left untreated in RPMI 1640 medium supplemented with 10% FCS. Six hours before the assay, brefeldin A (5  µg/mL) was added to induce the disassembly of the Golgi complex. After stimulation, cell viability was assessed by co-staining with a Zombi violet dye-BV42. Then, cells were washed with PBS-1%-FBS and stained for the surface markers. Fluorochrome conjugated antibodies CD3-A700, CD8-PerCP, CD4-BV510, CD62L-FITC, and CD44-PE (Biolegend) were used for the analysis of extracellular receptor molecules. After fixing with PBS-1%-FBS-1%-Saponine-4%-PFA, cells were permeabilized with PBS-1%-FBS-1%-Saponine and stained intracellularly using the fluorochrome-conjugated antibody IFN-γ–APC (Miltenyi Biotec, Bergisch Gladbach, Germany). Data were acquired by FACS analysis on a FACS Celesta Sorp platform (Becton Dickinson, Franklin Lakes, NJ, USA). Analyses of the data were performed using FlowJo software version 10.9 (Tree Star, Ashland, OR, USA). The number of lymphocyte-gated events was 10^5^. Lymphocytes were initially gated on the basis of their forward and side scatter properties. Then, CD8+ and CD4+ lymphocytes expressing IFN-γ were selected for the analysis. Naive T cells exhibit high levels of CD62L and low expression of CD44, whereas memory T cells are identified by high CD44 and low CD62L. Gating strategies used to identify CD8+ T-cell populations are shown in Fig. S1.

### Statistical analysis

Data were analyzed using GraphPad Prism version 8.0.1 (GraphPad Software, San Diego, CA, USA). Survival curves for each immunized group were compared to those of non-immunized mice in search of statistical differences using log-rank test. Data from the MVA growth kinetics, ELISPOT, ICS, PRNT_50_, and VP2 or VP7 ELISA assays were analyzed using Mann–Whitney non-parametric test. Comparisons of mean responses between groups in the viremia and RNAemia analysis were conducted by multiple t test analysis using the Sidak–Bonferroni method. Differences between groups regarding circulating cytokine levels were conducted by two-way ANOVA with a *post hoc* Tukey test for multiple comparisons. Correlation between optical densities of the ELISA test and PRNT_50_ was analyzed by nonparametric Spearman’s rank order correlation coefficient. A *P*-value lower than 0.05 was considered significant in all cases.

## Data Availability

The data and material used and analyzed during the current study are available from the corresponding author on reasonable request.

## SUPPLEMENTAL MATERIAL

Figure S1 jvi.01687-24-s0001.tif Gating strategy used to analyze EHDV-specific T-cell responses.

ASM does not own the copyrights to Supplemental Material that may be linked to, or accessed through, an article. The authors have granted ASM a non-exclusive, world-wide license to publish the Supplemental Material files. Please contact the corresponding author directly for reuse.
