# Supplementary figures and images for: Vaccine candidates based on MVA viral vectors expressing VP2 or VP7 confer full protection against Epizootic hemorrhagic disease virus in IFNAR(−/−) mice
Source: J Virol. 2024 Nov 7;98(12):e01687-24. doi: 10.1128/jvi.01687-24 (PMC11650994; doi:10.1128/jvi.01687-24)

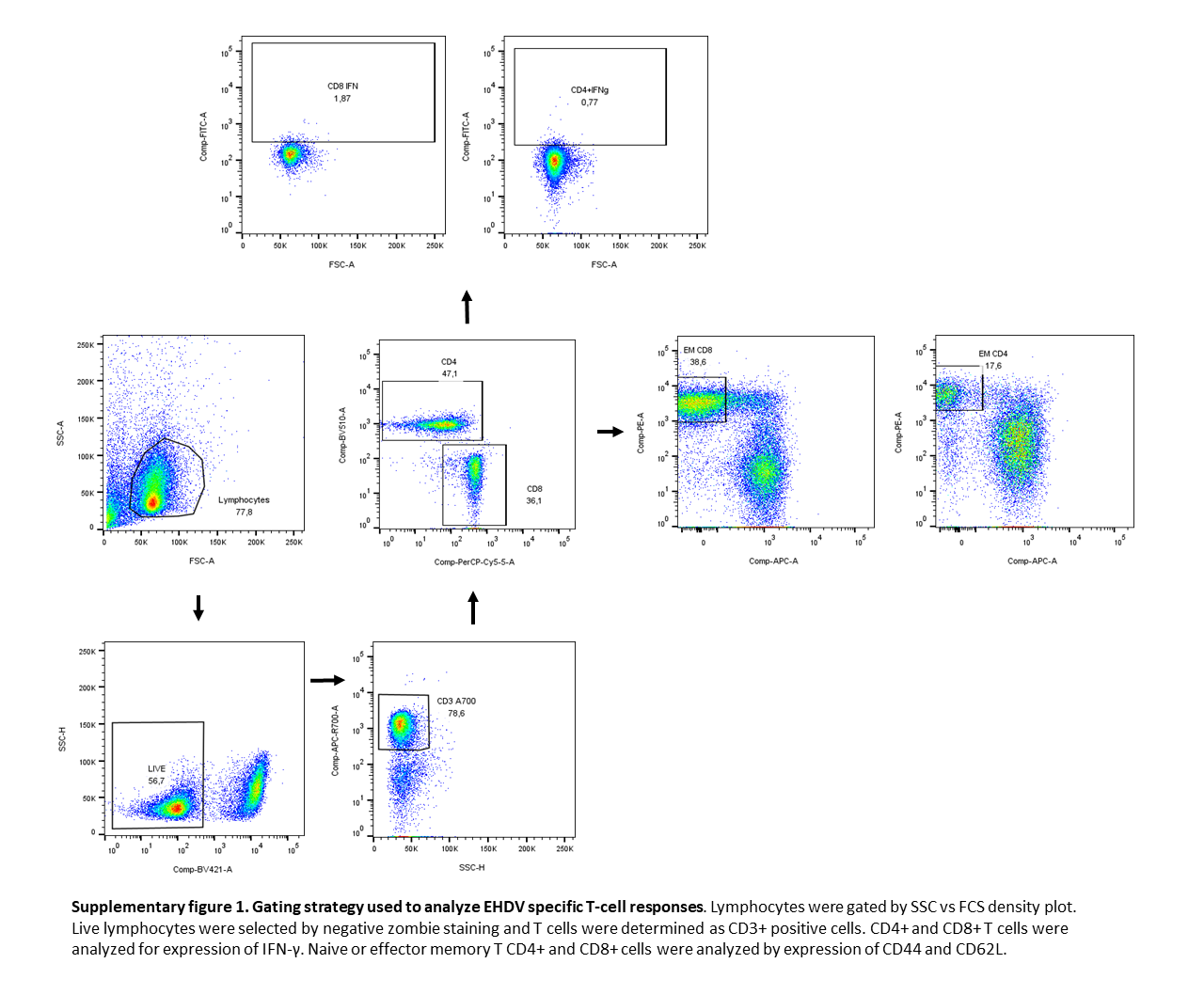

Supplement: Figure S1 — Gating strategy used to analyze EHDV-specific T-cell responses. [file jvi.01687-24-s0001.tif]
